# Review of the Microbiological Diagnostic Approaches of COVID-19

**DOI:** 10.3389/fpubh.2021.592500

**Published:** 2021-04-27

**Authors:** Ada Melo-Vallès, Clara Ballesté-Delpierre, Jordi Vila

**Affiliations:** ^1^Bachelor of Human Biology, Fourth Grade Student, Life and Health Sciences Faculty, Universitat Pompeu Fabra, Barcelona, Spain; ^2^ISGlobal Hospital Clínic–Universitat de Barcelona, Barcelona, Spain; ^3^Department of Clinical Microbiology, Centre for Biomedical Diagnosis, Hospital Clínic, Barcelona, Spain

**Keywords:** diagnostic testing, SARS-CoV-2, sensitivity, cross-reactivity, optimizing diagnostics

## Abstract

On March 12, the World Health Organization declared a pandemic following the exponential increase of SARS-CoV-2 cases. The rapid spread of the virus is due to both its high infectivity and the free circulation of unrecognized infectious cases. Thus, diagnostic testing is a key element to prevent further dissemination of the virus. Urged by WHO's call, laboratories worldwide have been working on nucleic acid tests protocols and immunoassays that became available, albeit poorly validated, within a comparatively short time. Since then, external studies evaluating these diagnostic tests have been published. The present study is a review of the COVID-19 diagnostic approaches, discussing both direct and indirect microbiological diagnoses. A compendium of the literature on commercial assays kits available to date is provided together with the conclusions drawn as well as RT-PCR protocols published by the WHO. Briefly, diagnostic accuracy varies according to time elapsed since symptom onset and evolves together with understanding of the COVID-19 disease. Taking into account all these variables will allow determining the most adequate diagnostic test to use and how to optimize diagnostic testing for COVID-19.

## Introduction

In December 2019, Chinese authorities reported an outbreak of cases of pneumonia of unknown etiology in Wuhan, China, of unknown cause. Characterization of the disease in a cluster of reported cases of pneumonia was associated with the spread of a novel coronavirus named SARS-CoV-2 (1). The rapid increase of Coronavirus Disease 2019 (COVID-19) cases, already being reported outside the Asian continent and evidence of human-to-human transmission, led to the declaration of a pandemic by the World Health Organization (WHO) on March 12th, 2020 (2, 3). Soon after the isolation of this new type of coronavirus (CoV) from bronchoalveolar lavage fluid, its viral genome sequence was released on the open access website virological.org (GISAID) (4, 5) to begin the development of diagnostic kits. Since then, a race to develop and distribute reliable diagnostic assays has been encouraged by World Health Organization (6).

SARS-CoV-2 is the seventh CoV known to infect humans and the third causing a severe acute respiratory syndrome, after SARS-CoV in 2002 and MERS-CoV in 2012 [the characteristics of the three CoV outbreaks are summarized in Table 1 (7, 8)]. Like SARS-CoV, the novel CoV belongs to the Betacoronavirus genus, subgenus *Sarbecovirures* (14).

**Table 1:** Characteristics of the three coronavirus outbreaks [information extracted from Wang et al. (7) from the study by Chen et al. study (8)].

	**SARS-CoV-2**	**MERS-CoV**	**SARS-CoV**
Outbreak date	December, 2019	June, 2012	November, 2002
Location of first detection	Wuhan, China	Jeddah, Saudi Arabia	Guangdong, China
Target receptor	ACE2	CD26	ACE2
Confirmed cases	119,791,453^a^	2,494	8,096
Confirmed deads	2,652,966^a^	858	744
Case fatality rate	3%	37%	10%
Ro	1.4–3.5^b^	<1	0.4–2.9^d^
Incubation period (days)	Range from 2 to 14^c^	5	2–7

a *Confirmed cases and deads updated on 16 March 2021 (9)*.

b *On January 23, the WHO estimated R0 to be between 1.4 and 2.5 (10). However, other preliminary studies such as that conducted by the Imperial College of London estimates R0 to be even higher at up to 3.5 (11)*.

c *SARS-CoV-2 Incubation period information was taken from the Centers for Disease Control and Prevention (CDC) webpage (12)*.

d *The SARS-CoV R0 value was taken from the “Consensus document on the epidemiology of severe acute respiratory syndrome (SARS)” published in 2003 by the WHO. From the initial phase of the epidemic, excluding superspreading events, R0 was estimated to be 2.9. Once control measures were implemented, the R0 value was reduced to 0.4 (April, 2003) (13)*.

To date, marking 12 months after the emergence of the pandemic, there have been more confirmed SARS-CoV-2 cases around the globe than MERS and SARS. The reasons for such a rapid spread include a high infectivity [studies assessing efficient SARS-CoV-2 cell entry mechanisms have uncovered that the novel CoV has a higher binding affinity to the human angiotensin-converting enzyme 2 (hACE2) than SARS-CoV (15, 16)], high transmissibility of the virus (17, 18), a longer incubation period, an efficient immune evasion (15) and a delayed response from government and institutions (19).

The measures adopted prior to physicians' advice to stay at home were not sufficiently effective to curb the virus, thereby explaining the high number of undiagnosed infectious cases that went unrecognized (18). Both the long incubation period and the large number of mild infections with limited or absent symptomatology contribute to a deficient early diagnosis. Moreover, it seems that patients can be highly contagious during the pre-syndromic period, which, in addition to the lack of adequate and sensitive diagnostic tests, has made case identification and isolation difficult (20, 21).

Diagnostic tests must be specific enough to discriminate SARS-CoV-2 from other CoV with which it shares a high degree of homology (22). Thus, sensitive and specific diagnostic testing is crucial to prevent further spread of the virus.

The present review aims to describe the current approaches to SARS-CoV-2 diagnosis including the different types of tests available and the current limitations and successful findings achieved during the previous 7 months of testing. Based on the current evidence and what has been learned to date about the infectivity and physiopathology of the virus, as well as the kinetics profile of the specific antibodies to SARS-CoV-2, we provide suggestions on how to optimize diagnostic testing for COVID-19 and the usefulness of antibody detection.

## Methodology

Urged by the call from the WHO to develop reliable diagnostic tools, laboratories worldwide have developed several commercial assays that have been granted an Emergency Use Authorization (EUA) by the Food and Drug Administration (23). Among all the assay tests released, it is important to distinguish direct from indirect assays (24). Direct tests detect the virus replication (active infection), and include real time reverse-transcriptase polymerase chain reaction (rRT-PCR) and antigen-based rapid diagnostic tests (Ag-RDTs). On the other hand, indirect tests search for host antibody response, detecting both active and past infections. These tests include Antibody-based Rapid Diagnostic Tests, usually by lateral flow (Ab-RDTs), and antibody-based diagnostic tests (Ab-DTs) using either enzyme-linked immunosorbent assays (ELISAs) or chemiluminescence enzyme immunoassays (CLIAs).

Since the beginning of the pandemic, 7 different in-house developed molecular assays protocols (RT-PCR) have been posted on the WHO website (25). These protocols have been elaborated by different investigation centers: Hospital Charité (Berlin, Germany) (26, 27), Hong-Kong University-Faculty of Medicine (HKU Med) (Hong-Kong, China) (28), Center for Disease Control of China (China CDC) (29), Institut Pasteur (Paris, France) (30), Center for Disease Control of USA (US CDC) (Atlanta, USA) (31), National Institute of Infectious Diseases (Tokyo, Japan) (32), and the National Institute of Health (Bangkok, Thailand) to guide laboratories involved in testing SARS-CoV-2 worldwide (33). The WHO has shown no preference for any of these assays and none has been endorsed or validated by the organization. Here we compare these different RT-PCR assays and summarize the finding in Table 2.

**Table 2:** Comparison of the different RT-PCR assays protocols published by the World Health Organization.

**Institute (Country)**	**Target gene**	**Throughout the text referred to as**	**Oligonucleotide**	**Sequence**	**Amplicon size (bp)**	**Polymerase**	**Thermocycler used in the reference publication**	**Volume of RNA extract**
Charité (Germany) (26, 27)	E^a^	E assay (Charité)	E_Sarbeco_F E_Sarbeco_R E_Sarbeco_P1	ACAGGTACGTTAATAGTTAATAGCGT ATATTGCAGCAGTACGCACACA FAM-ACACTAGCCATCCTTACTGCGCTTCG-BBQ	113	SuperScript^TM^ III Platinum® One-Step	Light Cycler® 480II (Roche) or Applied Biosystems ViiA ^TM^	5 μl
	RdRp^b^	RdRp assay	RdRp_SARSr-F	GTGARATGGTCATGTGTGGCGG	100	Quantitative	7 (ThermoFisher)	
		(Charité)	RdRp_SARSr-R	CARATGTTAAASACACTATTAGCATA		RT-PCR System		
			RdRp_SARSr-P1^c^	FAM-CCAGGTGGWACRTCATCMGGTGATGC-BBQ				
			RdRp_SARSr-P2^d^	FAM-CAGGTGGAACCTCATCAGGAGATGC-BBQ				
	N	N assay (Charité)	N_Sarbeco_F	CACATTGGCACCCGCAATC	128			
			N_Sarbeco_R	GAGGAACGAGAAGAGGCTTG				
			N_Sarbeco_P	FAM-ACTTCCTCAAGGAACAACATTGCCA-BBQ				
HKU Med (China) (28, 34)	N^a^	N assay (HKU Med)	HKU-N-F	TAATCAGACAAGGAACTGATTA	110	TaqMan Fast Virus	Applied Biosystems	4 μl
			HKU-N-R	CGAAGGTGTGACTTCCATG		Master mix	ViiA^TM^ 7	
			HKU-N-P	FAM-GCAAATTGTGCAATTTGCGG-TAMRA			(ThermoFisher)	
	ORF1b (nsp14)^e^	ORF1 assay	HKU-ORF1-F	TGGGGYTTTACRGGTAACCT	132			
		(HKU Med)	HKU-ORF1-R	AACRCGCTTAACAAAGCACTC				
			HKU-ORF1-P	FAM-TAGTTGTGATGCWATCATGACTAG-TAMRA				
China CDC (China) (29)	N	N assay (China CDC)	CCDC-N-F	GGGGAACTTCTCCTGCTAGAAT	99	Unspecified	Unspecified	Unspecified
			CCDC-N-R	CAGACATTTTGCTCTCAAGCTG				
			CCDC-N-P	FAM-TTGCTGCTGCTTGACAGATT-TAMRA				
	ORF1ab (nsp10)	ORF1 assay	CCDC-ORF1-F	CCCTGTGGGTTTTACACTTAA	119			
		(China CDC)	CCDC-ORF1-R	ACGATTGTGCATCAGCTGA				
			CCDC-ORF1-P	FAM-CCGTCTGCGGTATGTGGAAAGGTTATGG- BHQ1				
Institut Pasteur (France) (30)	RdRp IP2	RdRp-IP2 assay (Pasteur)	nCoV_IP2-12669Fw	ATGAGCTTAGTCCTGTTG	108	SuperScript^TM^ III Platinum® One-Step Quantitative RT-PCR System	Light Cycler® 480 (Roche)	5 μl
			nCoV_IP2-12759Rv	CTCCCTTTGTTGTGTTGT				
			nCoV_IP2-12696bProbe(+)	HEX-AGATGTCTTGTGCTGCCGGTA-BHQ1				
	RdRp IP4	RdRp-IP4 assay	nCoV_IP4-14059Fw	GGTAACTGGTATGATTTCG	107			
		(Pasteur)	nCoV_IP4-14146Rv	CTGGTCAAGGTTAATATAGG				
			nCoV_IP4-14084 Probe(+)	FAM-TCATACAAACCACGCCAGG-BHQ1				
	E^e^	E assay (Charité)	E_Sarbeco_F	ACAGGTACGTTAATAGTTAATAGCGT	113			
			E_Sarbeco_R	ATATTGCAGCAGTACGCACACA				
			E_Sarbeco_P1	FAM-ACACTAGCCATCCTTACTGCGCTTCG-BBQ				
US CDC (USA) (31)	N	N1 assay (US CDC)	2019-nCoV_N1-F 2019-nCoV_N1-R 2019-nCoV_N1-P	GACCCCAAAATCAGCGAAAT TCTGGTTACTGCCAGTTGAATCTG FAM-ACCCCGCATTACGTTTGGTGGACC-BHQ1	72	TaqPath^TM^ 1-Step RT-qPCR Master Mix, CG (Thermo Fisher)	Applied Biosystems^TM^ 7500 Fast (ThermoFisher)	5 μl
	N	N2 assay (US CDC)	2019-nCoV_N2-F	TTACAAACATTGGCCGCAAA	67			
			2019-nCoV_N2-R	GCGCGACATTCCGAAGAA				
			2019-nCoV_N2-P	FAM-ACAATTTGCCCCCAGCGCTTCAG-BHQ1				
	N^f^	N3 assay (US CDC)	2019-nCoV_N3-F	GGGAGCCTTGAATACACCAAAA	72			
			2019-nCoV_N3-R	TGTAGCACGATTGCAGCATTG				
			2019-nCoV_N3-P	FAM-AYCACATTGGCACCCGCAATCCTG-BHQ1				
	Human Rnase P	HRnaseP assay	RP-F	AGATTTGGACCTGCGAGCG	Unspecified			
		(US CDC)	RP-R	GAGCGGCTGTCTCCACAAGT				
			RP-P	FAM-TTCTGACCTGAAGGCTCTGCGCG-BHQ1				
National Institute of Infectious Diseases (Japan)^g^ (32)	N	N assay (N.I.Infectious Diseases)	NIID_2019-COV_N_F2 NIID_2019-COV_N_R2	AAATTTTGGGGACCAGGAAC TGGCAGCTGTGTAGGTCAAC	Unspecified	Unspecified	LightCycler96 system (Roche)	5 μl
			NIID_2019-COV_N_P2	FAM-ATGTCGCGCATTGGCATGGA-BHQ				
National Institute of Health (Thailand) (33)	N	N assay	WH-NIC N-F	CGTTTGGTGGACCCTCAGAT	Unspecified	Invitrogen superscript^TM^ III Platinum One-Step Quantitative	Unspecified	5 μl
		(N.I.Health)	WH-NIC N-R	CCCCACTGCGTTCTCCATT				
			WH-NIC N-P	FAM-CAACTGGCAGTAACCA- BQH1				

*FAM, 6-carboxyfluorescein; HEX, hexachlorofluorescein; TAMRA, tetramethylrhodamine; BBQ, blackberry quencher; BQH1, black hole quencher*.

*Table adapted from Etievant et al. study (35) and Vogels et al. study (36)*.

*Red Bold capital letters indicate degenerated nucleotides (W is A/T; R is G/A; M is A/C; S is G/C; Y is C/T)*.

a *Target used as a first screening tool*.

b *Target used for confirmation and further discrimination between SARS-CoV and SARS-CoV-2*.

c *Probe specific for SARS-CoV-2 detection*.

d *Probe specific for SARS-CoV-2, SARS-CoV and bat-SARS-related CoVs detection*.

e *Target used for confirmation*.

f *On March 15th, the N3 primer and probe was removed from the Diagnostic panel*.

g *Apart from developing the RT-PCR protocol, the National Institute of Infectious diseases (Japan) besides developing the RT-PCR protocol also developed a nested RT-PCR protocol, which is not presented in here*.

With regard to immunoassays and Ag-RDTs, website of the Foundation for Innovative New Diagnostics (FIND) lists all the SARS-CoV-2 tests commercially currently available or under development (37, 38). Diagnostic kits are submitted by the manufacturer itself or taken from publicly published information. High-speed production and the urgent need for diagnostic tests has resulted in the launching of poorly validated assays in the market (39). The present review only includes commercial kits fulfilling the inclusion criterion of diagnostic kits supported by published literature, tested independently from the manufacturer, and providing both sensitivity and specificity values. Sensitivity was assessed comparing test performance against a gold standard technique. The final selection was updated on 13th May, 2020 and is presented as supporting material in this review (Supplementary Tables 1–4).

## Direct Diagnostic Testing

### Molecular Assays Protocols Published by the WHO and Developed by Referral Laboratories

**Figure 1:**
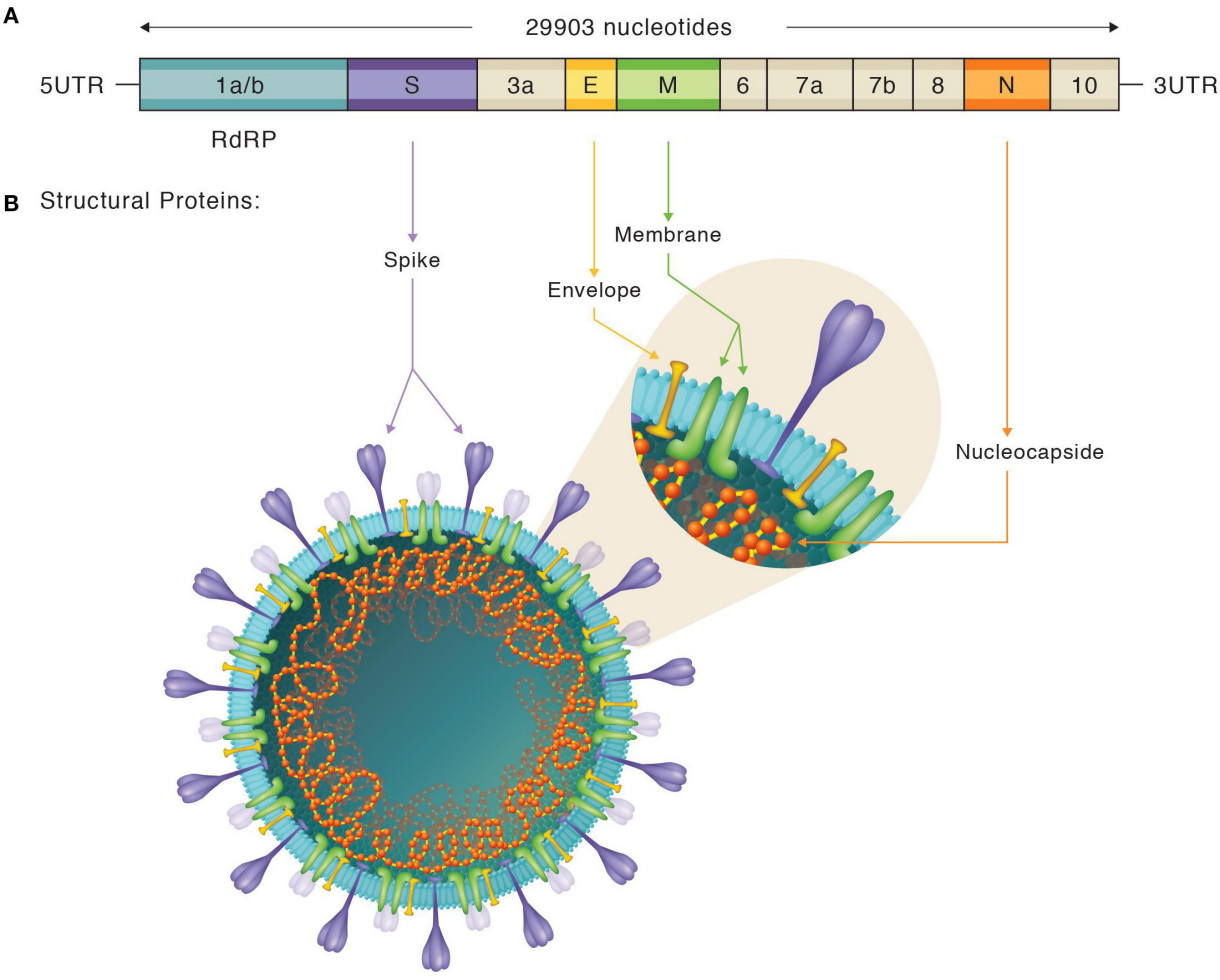
Depiction of SARS-CoV-2 genome and structure. **(A)** Organization of the SARS-CoV-2 genome. Both structural and non-structural proteins are represented. Figure adapted from both the Khailany et al. and Chan et al. studies (22, 42). **(B)** Illustration of SARS-CoV-2 structure, locating, and labeling all the structural proteins. Spike proteins subdomains are also shown.

To date, rRT-PCR is considered the gold standard technique for the diagnosis of SARS-CoV-2 infection since the symptomatology is non-specific and inconclusive, and other biological markers are non-exclusive of SARS-CoV-2 (40, 41). As SARS-CoV-2 is a positive-stranded RNA virus, reverse transcription into cDNA is needed prior to amplification. Genomic characterization of this novel CoV revealed conserved Betacoronavirus genome arrangement comprised from 5′ to 3′: the open reading frame (ORF) 1a/b [encoding for non-structural proteins (nsp)] and genes encoding structural proteins such as: the spike (S), the envelope (E), the membrane (M), and the nucleocapsid (N). Non-structural proteins are involved in transcription and replication, including the RNA-dependent RNA polymerase (RdRp) also named Nsp12. Additionally, the SARS-CoV-2 genome encodes for some accessory ORF proteins: ORF3a, ORF6, ORF7a, ORF7b, and ORF8 [(22, 42); see Figure 1].

The rRT-PCR protocol of Corman et al. (Charité) is the first to have been published and is one of the protocols most commonly implemented by laboratories worldwide (26, 27, 43). When virus isolates or samples from infected patients were not yet available, the Corman approach was based on both the close genetic relatedness to SARS-CoV and the use of synthetic nucleic acid technology. Based on the social media announcement of a SARS-related CoV and on the possibility of an increased sequence variability, an assay targeting the gene encoding the E protein was developed as a first screening tool (with wide sensitivity for detecting even phylogenetic outliers) followed by a confirmatory *rdRp* gene assay for further discrimination of SARS-CoV-2 RNA from SARS-CoV RNA (Table 2). Upon release of the novel CoV genome sequence, SARS-related virus sequences downloaded from GenBank were aligned with the SARS-CoV-2 sequence to confirm selected primer matching. The assay showed no cross-reactivity when tested with all endemic human CoV [NL63 (HCoV)229E, etc.] Shortly after the publication of the work of Corman, the European Virus Archive Global (EVAg) made available SARS-CoV positive controls and a panel of cell-culture RNA from different CoV available to check the specificity of the newly developed assays (44).

The same strategy was followed by Chu et al. (28) and HKU Med (34) (Table 2), who developed two-target rRT-PCR—primers against the ORF1b and N—sequence regions. The primers were intentionally made to be reactive to multiple viruses from the clade *Sarbecoviruses*, since there was still not enough information of the virus genetic diversity. When aligned with the SARS-CoV-2 genome sequences, which were gradually being posted on GISAID, the primers were confirmed to match perfectly. Sequence variations among genome targets were translated into degenerate nucleotides on the primers. With the exception of the novel SARS-CoV-2 and SARS CoV, no other *Sarbecoviruses* have ever been detected in humans (14). This affirmation, and the fact that the last reported human SARS case dates back to 2004, supports positive reported cases being attributed to SARS-CoV-2 (45). Amplification of the gene encoding for the N protein was found to be more sensitive than the ORF1b gene assay, suggesting that the first assay could be used as a screening assay using the latter as a confirmatory test.

Scientists from the Institute Pasteur, chose targeting two RdRp targets (IP2 and IP4) using the *E* gene assay from the protocol of Charité, which had just been published, as a confirmatory assay (30). The US CDC opted for the use of three primer-probe sets targeting three *N* gene encoding regions. The innovative strategy in this case was to use an additional primer set targeting the human RNase P gene. Failure to detect the RNase P gene would indicate poor biological sampling suggesting an invalid test result (31). Little is known about the other three protocols: the Chinese CDC protocol targets both the ORF1ab and N genes, while the Thailand protocol only targets the latter. The Japanese protocol uses pan-coronaviral primers that have worked in the past, and at the same time target multiple Spike proteins and Nucleocapsid regions, using both nested and rRT-PCR [(32); Table 2]. Overall, nucleic acid amplification tests targets so far include the N, the E, the S proteins, and the RNA-dependent RNA polymerase encoding genes (46).

### Sensitivity and Specificity Assessment of Primer-Probe Sets Published by the WHO

During the months that followed, WHO protocols were widely implemented in laboratories worldwide. Since then, some studies have assessed and compared the sensitivity and specificity of the different RT-PCR, reporting their limitations and assisting laboratories in their choice of protocol.

Although all primer-probe sets perform well when tested and can be used to detect SARS-CoV-2, there are some differences in regard to sensitivity: the RdRp assay (Charité) was found to have the lowest sensitivity [it only partially detected SARS-CoV-2 RNA for all 10–10^2^ viral RNA copies/μL concentrations while other assays were able to detect the RNA (Ct values <40)] (36). Etievant et al. also reported a worse sensitivity with the RdRp assay (Charité) compared to other assays when testing the lowest dilutions (35).

Vogels et al. reported that the E (Charité), the ORF1 (HKU Med), the N (HKU Med), the N (China CDC), the N1 (US CDC), and the N3 assays (US-CDC) were the most sensitive primer-probe sets, being able to detect SARS-CoV-2 at 1 and 10 viral RNA/μL (36). The study by Etievant et al. agrees with the primer-probes listed and also adds the RdRp-IP2 assay (Pasteur) and the RdRp-IP4 assays (Pasteur) (of note, the study by Vogels did not assess the Institute Pasteur's assays). The study by Etievant goes one step further, and while accepting that almost all the test are reliable for detection when used in clinical samples (excluding the RdRp and N assays by Charité and the US CDC N2 assay), they found that the N (China CDC), N1 (US CDC), N3 (US CDC), RdRp-IP2 (Pasteur), and the RdRp-IP4 assays (Pasteur) were the most sensitive, highlighting their low limit of detection, Ct values, and performance when testing different RNA concentrations obtained by serial dilutions (35).

The main difference between the Vogels and Etievant studies is the HKU primer-probes assessment. While Vogels states that the ORF1 assay (HKU Med) is one of the most sensitive assays, the Etievant study places it below the N1 (US CDC) and the N assays (China CDC). The discrepancy in sensitivity in the case of the ORF1 assay (HKU Med) may be because Vogels standardized PCR conditions for primer-probes comparisons and did not reproduce the protocols characteristics, unlike Etievant, who did follow different PCR conditions. While it's not clear whether this influences the sensitivity, Vogels reproduced a more realistic scenario since not all laboratories will be able to work within protocols conditions. Another possible explanation could be the use of different RNA extraction kits: Etievant extracted nucleic acid using the EMAG platform (Biomerieux, France) while Vogels used the MagMax Viral/Pathogen nucleic acid isolation kit (Thermofisher). Concerning specificity, background amplification was not observed in any of the nasopharyngeal swabs collected prior to the COVID-19 tested with primer-probe sets (36).

Although all the protocols have been considered reliable for achieving an accurate diagnosis, some irregularities have been noted. Etievant reported that the E (Charité) and the N2 assays (US CDC) were positive for all the dilutions tested including negative samples and controls. When analyzing amplicon size, both unspecific amplification and contamination were noted. It has been previously reported (47) that some other laboratories have received Corman et al. (26) *E* gene and *RdRp* gene tests cross-contaminated with synthetic controls.

### Mismatches in Primer Binding Regions May Result in a Decreased SARS-CoV-2 Detection

With regard to the RdRp assay (Charité), low performance (35, 36), and suspected nucleotide substitutions on primer annealing genome regions have been reported. Vogels had calculated the accumulated genetic diversity up to 22 March 2020 using the 992 SARS-CoV-2 genome sequences available at GISAID (36). The mutagenic capability of RNA virus depends on RdRp fidelity among other factors as this enzyme is implicated in both replication and proofreading activities. Several mutations that may compromise its activity have already been found on the RdRp encoding region, being one of the possible causes of the sudden increase in the number of mutations outside the Asian continent with the spread of the pandemic (48). Characterization and tracing of mutations can be valuable for designing and reviewing diagnostic assays. Vogels detected nucleotide mismatches in 12 primer-probe sets (belonging to the 7 different molecular assays protocols posted by the WHO) that have occurred in at least two of the 992 SARS-CoV-2 genomes [primer-probe mismatches referred to are listed in Figure 6 of (36)]. The most noted mismatch is the CT substitution (on genome position 15.519) present in 990 of the 992 SARS-CoV-2 genomes (36). The primer *RdRp_SARSr* (Charité) contains a degenerate nucleotide (S) on the corresponding nucleotide (12th position of the primer) that is intended to pair with G or C (nucleotides found at this position on SARS-CoV and bat-SARS-related CoV genomes). The degenerated nucleotide was purposely added to help the primer anneal to SARS-CoV and bat-SARS-related CoV genomes (26). This substitution compromises primer annealing to the SARS-CoV-2 genome, thus explaining the poor sensitivity reported. Another variant detected was TC substitution on 39 genomes (at genome position 28.688) compromising *2019-nCoV_N3* (US CDC) forward primer annealing (Table 2). This was detected and the primer was removed from the diagnostic panel (31). The three nucleotide substitutions (GGG → AAC) at genome positions 28.881–28.883 in 12.7% of the SARS-CoV-2 genomes, comprising the *CCDC-N* (US China) primer target sequence, do not seem as critical since their placement upstream on the primer does not seem to compromise their capability to anneal and amplify (36, 49).

With the posting of the SARS-CoV-2 sequences from the beginning of the pandemics, GISAID has updated the information regarding the variability of the primer target sequences. The last update in March 2020 reported that the N (from China CDC and HKU Med) and the N3 assays (US CDC) had the highest rate of mutations in the 3′ end of the primer (defined as the last 5 nucleotides of the primer sequence). These mutations could partially compromise sensitivity (50).

### Avoiding False Negatives When Performing the RT-PCR Test

Even though RT-PCR is considered the gold standard technique to diagnose COVID-19, recurrent notifications of false negatives have cast doubts on this methodology. Although most false negatives can be associated with poor sample collection usually of nasopharyngeal or oropharyngeal swabs, false negatives can also be attributed to the technique itself, aside from personnel skill, and to a lack of knowledge of the characteristics of the virus (virus shedding route and viral load kinetics), which is needed to address where and when to detect the virus (51).

Up to now, SARS-CoV-2 detection has consisted in first targeting a wide range of members of the *Sarbecoviruses* family, and second, in using specific probes for the further discrimination of SARS-CoV-2. This was done on purpose since at the time the first molecular assays were developed, little was known about the genetic diversity of the virus. Targeting a broad range of specimens implies designing primers that recognize a range of variability in detriment of specificity, thereby resulting in a higher number of false negatives. New approaches have attempted to identify SARS-CoV-2 directly, by establishing globally conserved targets, such as the COVID-19-nsp2 or the COVID-19-RdRp/Hel assays (52, 53).

Concerning the shedding pattern, SARS-CoV-2 viral load kinetics differs from that of SARS-CoV, resembling the influenza virus and also peaking soon after symptom onset (54, 55). Transmission of SARS-CoV occurs several days after symptom onset: the peak viral load is reached 7–10 days after illness onset (5 × 10^5^ copies per swab) (56) and by day 15, viral levels are lower than on admission (57). Symptoms appeared after 2–7 days of incubation: therefore, isolation measures are very effective, since by the time symptoms appear and diagnosis has been determined, the subjects are still not at the peak of infectiousness. With SARS-CoV-2, transmission occurs earlier in the course of infection, when symptoms are either absent or mild (55). The highest viral load is reached 5 days before illness onset (7,11 × 10^8^ copies per throat swab), the same time that it takes for symptomatology to appear. By the time patients are admitted to hospital and testing is performed, the shedding peak in the upper respiratory tract has already been reached, and possible contagions have already occurred (56). The viral load starts decreasing from the 5th day after illness onset. After this point the chances of detecting viral load are progressively smaller—and the possibility of a false negative is higher. In this case, if the patient was diagnosed in an advanced state of the illness, immunological assays would likely be a better option than repeatedly performing nucleic acid tests.

Finally, successful virus isolation from throat swabs, as well as identification of viral subgenomic messenger RNAs provided proof of virus replication in the upper respiratory tract (56). Another study described nasal swabs as the optimal type of sample for SARS-CoV-2 detection, followed by throat swabs, which are more problematic in mild cases or samples collected beyond 15 days after symptom onset (58). Evidence of multiple SARS-CoV-2 shedding routes and body site specific virus replication depending on the severity have been published (58, 59).

### An Improved Version of the Accurate Molecular Testing

Both the low turnaround time and the impossibility of processing a large number of samples at the same time are the major drawbacks of molecular testing approaches. For over a decade, the Cepheid (Sunnyvale, USA) has been working on the innovation of the molecular diagnosis, developing an automated molecular testing platform named GeneXpert System, which allows point-of-care testing (60). Following the announcement of the novel SARS-CoV-2, Cepheid launched a SARS-CoV-2 molecular diagnostic test (the Xpert Xpress SARS-CoV-2 cartridge) that was granted EUA on March 21st. This technology works as follows: the sample is added into the cartridge and the latter is loaded into the GeneXpert System which automatedly runs samples and generates results within 30 and 40 min depending on whether the result is positive or negative. A study assessing the Xpert Xpress SARS-CoV-2 assay conducted in the Netherlands has recently been published, reporting equal performance compared to in-house RT-PCR (61). The WHO has stated that the assay is “well-suited to complement a wider testing strategy.” In addition, one of the major advantages is that the GeneXpert System is already available and distributed in some countries, as it is used as a diagnostic assay for tuberculosis and to test drug-resistant specimens (62). Another example is the Qiagen product (Venlo, the Netherlands) named QIAstat-Dx Respiratory SARS-CoV-2 panel, that received EUA on March 31st. This panel also uses a cartridge that detects and differentiates among 22 respiratory targets, including the SARS-CoV-2, by targeting both the *ORF1ab* and the *E* genes (63). DiaSorin Molecular LLC has also developed and manufactured the Simplexa TM COVID-19 direct kit that is run on the thermocycle LIAISON® MDX. The sample, which undergoes no extraction step, is loaded into an amplification disc (into which 8 different samples can be run at the same time). The assay targets both the ORF1ab and the S encoding regions (64, 65). The DiaSorin Simplexa product has demonstrated good performance, being slightly less sensitive than the Cepheid Xpert Xpress SARS-CoV-2 assay (66). Lastly, the Roche and the Hologic platforms named Cobas 6800 and Panther® system, respectively, have been widely implemented in microbiological laboratories. These automated systems also allow integrated extraction, amplification and detection of specimens. They offer a higher throughput, and a shorter hands-on time, thereby being less demanding (67, 68).

## Indirect Diagnostic Testing

### Basis of Indirect Testing and Rapid Diagnostic Tests

Indirect testing relies on the presence of host antibodies. SARS-CoV-2 antibody response has proven to be similar to that seen against many other acute viral infections (41, 69). IgM is the first immunoglobulin (Ig) to develop after antigen exposure, being an indicator of the early phase of infection, while IgG only appears at a later phase. IgM response is characterized as being more active during the first days after the onset of infection and then declining, while IgG levels increase and remain high for a much longer period of time (40). Despite showing a high activity, IgM is known to have a lower affinity compared to IgG (70). At the time of writing, several studies have shed light on host antibody response, as the value of diagnostic testing depends heavily on the understanding of response. The COVID-19 serological assays currently available target either IgM or IgG, or both, or IgA or total antibodies and are shown in Table 3.

**Table 3:** Commercial tests performance grouped by antibody detected and antigen used to.

**Type of test**	**Antibody detected**	**Antigen used for detection**	**Sensitivity**	**Specificity**	**Company**	**References**
ELISA	Total antibodies (Ab)	Receptor Binding domain	93.10%	99.10%	Beijing Wantai Biological Pharmacy Enterprise Co., Ltd. (China)	(41)
			93.00%	100.00%		(71)
			97.50%	100.00%		(72)
	IgM	Nucleocapsid protein	77.30%	100%	Zhuhai Livzon Diagnostics Inc. (China)	(73)
			68.20%	100.00%	Zhuhai Lizhu Reagent Co., Ltd. (China)	(69)
			46.10%	82.00%	Guangzhou Darui Biotechnology Co., Ltd. (China)	(74)
		Receptor Binding domain	82.70%	98.60%	Beijing Wantai Biological Pharmacy Enterprise Co., Ltd. (China)	(41)
			77.10%	100.00%	Beijing Hotgen Biotech Co., Ltd. (China)	(69)
			92.50%	100.00%	Beijing Wantai Biological Pharmacy Enterprise Co., Ltd. (China)	(72)
	IgG	Nucleocapsid protein	83.30%	95.00%	Zhuhai Livzon Diagnostics Inc. (China)	(73)
			64.70%	99%	Beijing Wantai Biological Pharmacy Enterprise Co., Ltd. (China)	(41)
			70.10%	100%	Zhuhai Lizhu Reagent Co., Ltd. (China)	(69)
			23.00%	100%	Guangzhou Darui Biotechnology Co., Ltd. (China)	(74)
			88.80%	100%	Beijing Wantai Biological Pharmacy Enterprise Co., Ltd. (China)	(72)
		Receptor Binding domain	74.30%	100%	Beijing Hotgen Biotech Co., Ltd. (china)	(69)
		Spike protein subdomain 1	67%	96%	Euroimmun Medizinische Labordiagnostika (Germany)	(71)
		Nucleocapsid protein and pike protein subdomain 2	88%	97%	Mologic Ltd. (UK)	(39)
	IgA	Spike protein subdomain 1	93%	93%	Euroimmun Medizinische Labordiagnostika (Germany)	(71)
LFIA	Total antibodies (Ab)	Receptor Binding Domain	97.5%	95.2%	Beijing Wantai Biological Pharmacy Enterprise Co., Ltd. (China)	(72)
	IgM	Receptor Binding domain	88.80%	98.10%	Beijing Wantai Biological Pharmacy Enterprise Co., Ltd. (China)	(72)
		Unspecified	43.20%	98%	Artron Laboratories Inc. (Canada)	(11)
			57.10%	100%	Zhuhai Livzon Diagnostics Inc. (China)	(73)
			55.80%	–		(75)
	IgG	Nucleocapsid protein	86.30%	99.50%	Beijing Wantai Biological Pharmacy Enterprise Co., Ltd. (China)	(72)
		Unspecified	14.40%	100%	Artron Laboratories Inc. (Canada)	(11)
			81.30%	100%	Zhuhai Livzon Diagnostics Inc. (China)	(73)
			54.70%	–		(75)
	IgM-IgG	Receptor Binding domain	30%	89%	Jiangsu Medomics Medical Technologies (China)	(99)
			88.66%	90.63%		(17)
		Unspecified	82.40%	100%	Zhuhai Livzon Diagnostics Inc. (China)	(76)
			90%	100%	Dynamiker Biotechnology (China)	(71)
			90%	100%	CTK Biotech (USA)	
			93%	100%	AutoBio Diagnostics (China)	
			83%	100%	Artron Laboratories Inc. (Canada)	
			18.40%	91.70%	Vivachek Biotech (China)	(21)
			88.90%	100%	Hangzhou Alltest Biotech Co., Ltd. (China)	(77)
CLIA	Total antibodies (Ab)	Receptor Binding domain	96.30%	99.30%	Xiamen InnoDx Biotech Co., Ltd. (China)	(72)
	IgM	Nucleocapsid protein and spike protein	48.10%	100%	Shenzhen YHLO Biotech Co., Ltd. (China)	(78)
			100%	97.33%		(59)
		Receptor Binding domain	86.30%	99.30%	XIamen InnoDx Biotech Co., Ltd. (China)	(72)
	IgG	Nucleocapsid protein and spike protein	88.90%	90.90%	Shenzhen YHLO Biotech Co., Ltd. (China)	(78)
			100%	99.56%		(59)

The development of rapid diagnostic tests (RDTs) began a short time after the outbreak of SARS-CoV-2, pursuing point-of-care (POC) diagnostic goals, which provide results “*at the time and site of an encounter*.” RDTs have proven to be effective for detecting other pathogens in the past (79). The Ab-RDTs included in the present review are based on the lateral flow immunoassay (LFIA) technology which, in turn, is based on the capillary migration principle. Briefly, sample targets flow along a membrane and bind to their matching antibody at the test line, providing a visual result (40, 80). In addition, fluorescent detection has also been developed. Compared to molecular assays, RDTs are less expensive, easier to perform, faster, do not need qualified personnel, and sample collection carries a lower risk of exposure. On the other hand, they have shown a considerably lower sensitivity and specificity (11, 39, 73).

Besides Ab-RDTs, ELISAs, a well-established type of immunoassay technique, and CLIAs have also been developed and are included in the present review (Supplementary Tables 1, 3). ELISAs and CLIAs provide quantitative data while Ab-RDTs only give qualitative results.

### Assessment Based on External Evaluation of Serological Assays

The heterogeneity of the testing conditions among the studies included such as the different number of days since symptom onset during sample collection and the different number of patients in which testing was performed, prevents the pooling of data to perform statistical analyses. In addition, some of the articles here reported data that have not yet been peer-reviewed, due to the recent onset of the pandemic. Therefore, this systematic search aims to be a compendium of the currently available literature on the commercial kits to test SARS-CoV-2 listed on the FIND website. Conclusions drawn by the different studies are collected and compared, with advice on which features provide better results. At present, FIND is evaluating some of the commercial immunoassays listed under the manufacturer's request, using a standardized independent protocol (81); with the objective of providing impartial data to guide laboratories in their choice of immunoassay. Until the FIND report is available, this review intends to provide recommendations based on the data available.

The final selection of articles included in the present review comprised 18 articles testing 7 different commercial ELISAs, 2 different commercial CLIAs, 9 different commercial Ab-RDTs based on LFIA technology, and 1 Ag-RDT (see Supplementary Tables 1–4). Some commercial assays are tested in more than one article, thus allowing comparisons of performance.

The tests differ, among other aspects, in the laboratory technique, the antigens used for antibody detection, and the type of antibody targeted. In addition, negative COVID-19 specimens used to test kit specificity across studies have a variable origin. Some studies assessed specificity testing in samples collected from healthy individuals prior to the SARS-CoV-2 outbreak, while others used samples from subjects with a negative COVID-19 result. It is important to note that almost none of the Ab-RDTs manufacturers provide information on which antigen was used for antibody detection. Finally, all the studies, with the exception of one, agree with the use of RT-PCR as the gold standard method for comparing test sensitivity.

The specificity of all the commercial immunoassays was generally very satisfactory, while the sensitivity was far from adequate. The tests showing the best performance, according to sensitivity values, were ELISAs, followed by CLIAs and finally Ab-RDTs (Table 3), although even the best commercial assays missed a number of false negatives. A test showing high performance according to specificity, results in an accurate positive predictive value (PPV) when applied to a high prevalence scenario. However, as infection incidences decline, the PPV decreases as well, resulting in an equal number of true and false positives (24). According to the current kits, which have a less than perfect specificity, and with the upcoming scenario of a low prevalence endemic infection, many more infectious cases will be missed (82).

Most of the studies evaluated sensitivity and specificity separately for IgM and IgG, with IgG mainly performing better than IgM. In scenarios in which this was not the case, the results are attributed to the difference in time of sample collection from symptom onset. A possible explanation would be that IgM detection covers a narrower phase of the infection time course than IgG. IgM appears earlier but also fades first, while IgG persists (40). Another theory could be the lower specificity attributed to IgM (70, 74). Some studies have reported an additional sensitivity value either testing total antibodies or considering a positive result if either of the two Igs was positive [(41, 69, 71, 75, 77); Xiang et al., 2020b]. Likewise, a higher sensitivity value was obtained when taking into account the two immunoglobulins together. The search for either of the two Igs covers a broader phase of the infection, increasing test sensitivity; Nonetheless, testing IgM and IgG separately is a better option than targeting total antibodies, as Igs titers provide valuable information of the course of the disease. Apart from IgG and IgM, only one study searched for IgA. The Euroimmun IgA ELISA showed both a low sensitivity and specificity, being more prone to cross-react with negative sera [Supplementary Table 1; (71)].

Finally, the ELISA immunoassay studies reporting the best performance used a double sandwich assay instead of a capture or an indirect ELISA (41, 71).

Whether test performance is affected when tested in milder COVID-19 cases remains unknown (71). Most of the studies included in this review tested commercial kits in severe COVID-19 cases that attended hospital in whom RT-PCR was performed.

Overall, Ab-RDTs are far from reliable in terms of sensitivity and specificity. Regardless of how attractive point-of-care diagnosis is, at present it cannot compensate for its poor performance. However, future improvements in these aspects, will make Ab-RDTs a promising solution for large-scale screening.

### Being Immunogenic and Avoiding Cross-Reactivity: Two Features Pursued by Candidate Antigens

Serological assays rely on the recombinant antigen with which they are coated. The antigen chosen must not only be immunogenic to ensure a high sensitivity but must also comprise specific epitopes to avoid cross-reactivity. In SARS-CoV, the N and the S proteins were found to be the dominant immunogenic antigens (83). This previous knowledge and the certainty that the novel CoV shares a high degree of similarity with SARS-CoV (82% of nucleotide identity) and presents the same structural proteins including S and N (22), makes the use of these two antigens promising in protein-based serological assays for detecting antibodies against SARS-CoV-2.

The N protein, is one of the major structural viral proteins and is involved in the transcription and replication of the genetic information of the virus and further encapsulation and packaging of the virions (69). It is a small, non-glycosylated protein, that is easy to clone and purify (Figure 1). During the SARS-CoV outbreak, N-protein based serological tests reported a high sensitivity paired with a low specificity, with a high rate of false-positive results (84). The same tests showed cross-reactivity among different known human CoVs (85) and autoantibodies in autoimmune diseases (86).

The other major immunogenic candidate is the S protein, a transmembrane glycosylated protein forming homotrimers that mediate CoV entry into the host cells (87). The S protein comprises two subdomains: S1, involved in specifically binding to the ACE2 receptor, and S2, responsible for membrane fusion. In turn, the S1 subdomain is made up of an N-terminal (S1^A^) domain and a receptor binding domain (RBD) [(14, 88); Figure 1]. The S protein is much longer than the N protein, and thus, it is difficult to obtain in full-length, and presents glycosylation sites. Denaturalized or non-glycosylated forms might modulate antibody recognition, leading to false-negative results (84).

In the years following the SARS-CoV outbreak, there was controversial literature as to whether the majority of neutralizing antibodies were directed against the N or S protein. Buchholz et al. reported that neutralizing antibodies against S proteins conferred protection and ultimately prevented host cell infection (89, 90). On the contrary, when studying the antibody response of SARS-infected individuals, Leung et al. observed that the N was the most frequently target, followed by the S (91).

The SARS-CoV-2 pandemic has revived the same doubts concerning which antigen protein is the most adequate for detecting antibodies. To date, the kits available include the use of the RBD, and the N, or the S protein (Table 3).

### Most Promising Antigen Used for SARS-CoV-2 Detection

The Alphacoronavirus, another CoV genus, is known for being responsible for a number of seasonal common cold cases every year. Consequently, a large proportion of the population possesses antibodies against one of the four human endemic CoVs (92). Phylogenetic closeness and conserved immunogenic proteins might result in cross-reactivity and false-positive results when testing for SARS-CoV-2, as seen previously with SARS-CoV.

Okba et al. tested several in-house and commercial assays that used different recombinant antigens showing that the S1 subdomain is more appropriate for SARS-CoV-2 detection than the S2 (or, by extension, the full-length S protein), as the latter is more conserved in CoV (88) (percentage amino acid identity of coronavirus conserved proteins to the novel coronavirus proteins can be found on Okba et al. work). N protein-based serological tests also proved to be sensitive, even though the N antigen appears to be more conserved than the S protein. All the assays tested showed no cross-reactivity among other CoV except for SARS-CoV sera, possibly due to the highest degree of similarity Okba et al. (88). The authors do not consider this an issue since the last case of SARS reported dates back to 2005 (45) and SARS-CoV specific antibodies are no longer detectable in serum of SARS-infected subjects that had been tracked for 6 years (93).

A study comparing commercial test performance between a rN-based ELISA (Zhuhai Lizhu Reagent Co., Ltd.) and a rS(RBD)-based ELISA (Beijing Hotgen Biotech Co., Ltd.) was conducted by Liu et al. (69) (Supplementary Table 1). The results showed that the rS-based ELISA was more sensitive for detecting IgM antibodies, with the S antigen being more immunogenic. Early response against the S protein compared to the N antigen is given as a possible explanation. However, previous literature on SARS-CoV disagree with this, and suggest that antibodies against the S protein are developed later in the infection (94).

Amanat et al. developed two in-house ELISA versions coated with the S protein antigen: the first with the full-length protein, the second with only the RBD. The full-length version showed stronger reactivity possibly due to the larger number of epitopes that encodes the larger version of the antigen (95). Stronger reactivity associated with a larger antigen fragment has also been described by Lassaunière et al. (Supplementary Table 1), who compared two commercial ELISA kits: the RBD-based ELISA from Beijing Wantai Biological Pharmacy Enterprise Co., Ltd and the S1-based ELISA from Euroimmun Medizinische Labordiagnostika (71). The latter showed cross-reactivity to serum containing HKU1 and adenovirus antibodies, suggesting than epitopes outside the RBDs are prone to inducing cross-reactivity.

RBD has demonstrated to be especially variable, varying more than the S2 subdomain or even than the N protein, being the major differentiator between the SARS-CoV-2 and the remaining CoVs, and is thus, becoming a promising antigen (14, 22, 88). While some studies have reported encouraging results with the use of SARS-CoV S-directed polyclonal antibodies to inhibit SARS-CoV-2 entry into host cells (which seems to be a contradiction), the explanation lies in the fact that successful antibodies do not target the ACE2 binding site within the SARS-CoV-2 RBD, but rather the S2 subunit (16, 87, 96). Even if the RBD has proven to be specific enough to avoid cross-reactivity, further studies are needed to ascertain whether it is immunogenic enough in comparison with the N-protein. Using two different antigens to check for antibodies might be a solution to avoid false negatives.

### Sensitivity Performance Varies Depending on Time Since Symptom Onset

The heterogeneity of the sensitivities reported across immunoassays is too high to be attributed only to the type of antibody detected or the antigen used in the assay. It should be noted that the number of days since symptom onset at which samples were collected to test commercial immunoassays vary across studies (Supplementary Tables 1–4). Indeed, in some cases, the authors decided to stratify samples according to time elapsed since illness onset, thus reporting different sensitivity values, and as expected, the performance was better with each passing day, as expected (69, 71). The increasing sensitivity depending on time determines the underlying growth profile of specific antibodies to SARS-CoV-2. The values in the table show that after day 8 from disease onset, antibody sensitivity exceeds that of RNA testing (41), suggesting that the decision of the type of diagnosis test should be based on time elapsed since illness onset.

Considering that RT-PCR sensitivity is a dynamic value across time, it raises the question as to whether to use this technique as the gold standard method to compare test sensitivity. If RNA testing is performed in the second week after illness onset or later, the sensitivity falls, missing false-negatives, and thereby providing misleading immunoassays sensitivity values.

## Optimizing Diagnostic Testing

It is essential to understand the difference between a diagnostic test and a test designed to study immunization status. The goal of the latter, which will not be discussed below, is the search for past infections, while the former searches for active infections (24). Diagnosis aims to detect subjects carrying active infections for further isolation and preventing the spread of the virus among contacts, together with providing early treatment. In order to achieve this goal, the appropriate tests should look at both antibodies and antigens.

Likewise, it is important to define what is meant by a screening test. Due to their easy-to-use and low turnaround condition, screening tests can cover a wide range of the population, which explains why they are so appealing for tracking infectious diseases and can be used to either diagnose unrecognized SARS-CoV-2 infectious cases or to determine seroprevalence levels among the population (24). If used as a diagnostic test, screening tests normally require further confirmation due to the low sensitivity and specificity they present (97).

**Figure 2:**
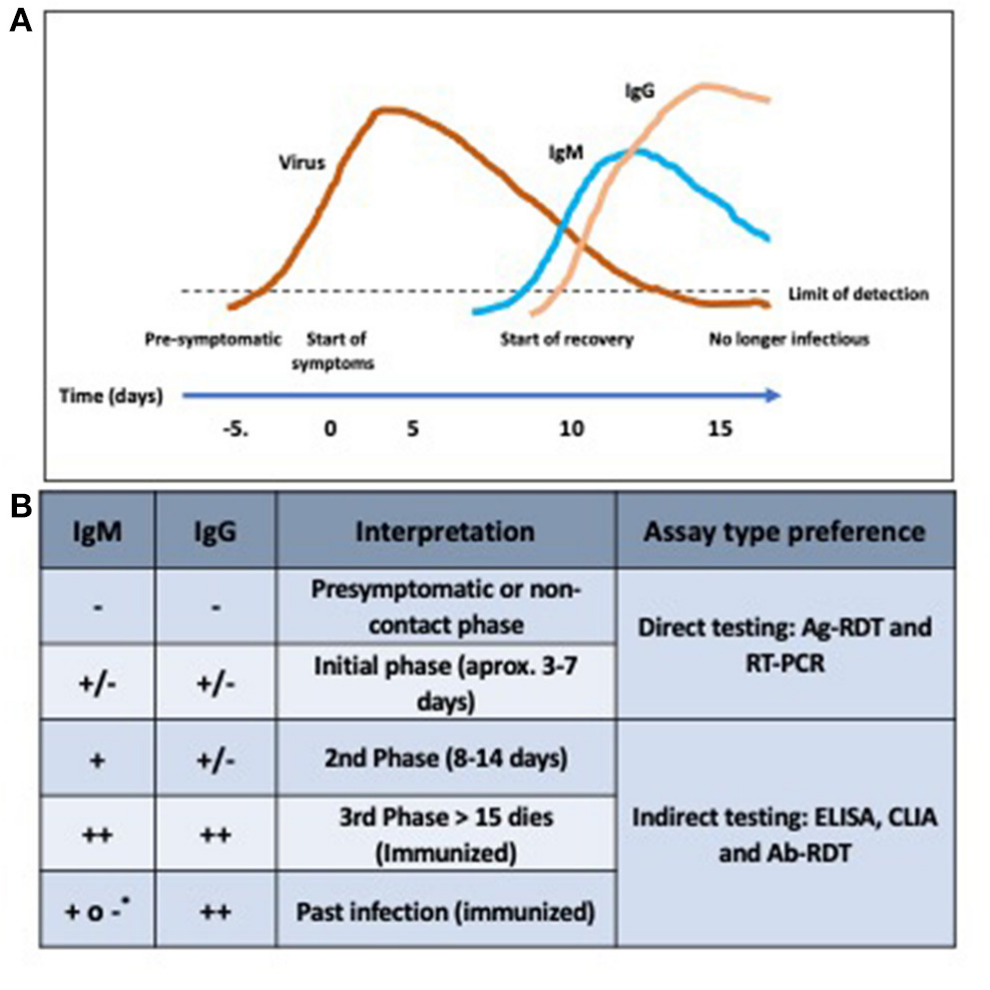
The course of infection according to antibody lecture. **(A)** Antigen shedding and antibody kinetics profile along the course of infection. Antigen levels persist despite the appearance of antibodies. Thus, active infection can be diagnosed either by either antigen or antibody detection. **(B)** Interpretation of the presence of antibodies. *IgM+ can appear as in some cases IgM can last overtime.

Optimizing diagnostics entails improving the choice of the diagnostic test based on the time since illness onset and understanding of COVID-19 disease, which in turn determine whether to look for antibodies or antigens (98). Furthermore, when performing diagnostic testing it is necessary to learn how to read and interpret the results [(99); Figure 2].

Viral antigens and genome are specific markers of the virus that precede both symptomatology and immunoglobulin response (98). In the pursuit of an early diagnosis, direct testing becomes the first option. Unlike the many Ab-RDTs available, there is only one Ag-RDT listed on the FIND website (manufactured by the Bioeasy Biotechnology Company) that is backed by two independent evaluations (98, 100).

This data should not be misinterpreted, as they exist other Ag-RDT authorized by other sources besides the one just mentioned. As stated at the beginning, the present review only includes commercial kits fulfilling the inclusion criterion of diagnostic kits supported by published literature, tested independently from the manufacturer, and providing both sensitivity and specificity values. Following such a strict criterion resulted in the inclusion of only one Ag-RTDs following FIND data search. However, since July 2020, the Food and Drug Administration has conceded 10 Emergency Use Authorizations for SARS-CoV-2 Ag-RDT. Approved Ag-RDT, listed in the Food and Drug Administration webpage, have been developed by manufacturers such as Quidel Corporation, Becton Dickinson and Company (BD), LumiraDx UK Ltd., among others (101). Besides, another six Ag-RDT have been approved by Japan's Pharmaceutical and Medical Devices Agency (102). Recently, several publications (103–105) have reported the sensitivity and specificity of Panbio COVID-19 Ag test with overall sensitivities that went from 73.3 to 91.7%, whereas when restricting the Ct to <32 the sensitivity went up to between 86 and 98%.

Currently, the World Health Organization (106) recommends using Ag-RDTs to respond to suspected outbreaks of COVID-19 in remote settings, institutions and semi-closed communities; to monitor trends in disease incidence in communities or when there is a widespread community transmission. In addition, they considered to test asymptomatic contacts of cases even if the Ag-RDT is not specifically authorized for this use. This last point is controversial and more studies should be carried out in order to support this statement.

If it were not for the poor accuracy reported, Ag-RDTs would have been a suitable option for large-scale detection of infectious cases before the appearance of symptoms, since despite being highly sensitive, RT-PCR molecular tests have a slower turnaround time, and are therefore less suitable for population screening. Consequently, RT-PCR molecular tests have been the standard technique to diagnose and screen contacts among reported infectious cases.

Active infections can also be diagnosed by detecting antibodies against SARS-CoV-2. A negative nucleic acid test result does not necessarily exclude the possibility of being infectious. Likewise, a positive immunoassay test result does not necessarily translate into antigen clearance. It has been demonstrated that RNA levels persist despite the appearance of antibodies (41). This raises many concerns related to discharge criteria (51, 107). A diagnosis based on antibody detection, however, is constrained by the time-dependent appearance of Igs (71). Antibodies against SARS-CoV-2 are detected from the middle stage of the course of infection (73). More specifically, IgM overtakes the detection cut-off value at day 9 after onset, and peaks at day 18. On the other hand, IgG is produced at some point between day 9 and 12 after onset, showing a rapid surge by day 15, and continuing to rise more steadily until day 39 (108). Another study reported that seroconversion times for total antibodies, IgM and IgG are 11, 12, and 14 days, respectively (41). Comparison of seroconversion rates between non-critical and critical cases showed no significant differences (41). Since little is known about the asymptomatic and autoimmune antibody kinetics profile, no advice is given on how to optimize the diagnosis of these groups of subjects based on the immune response.

Immunoassays are considered as a complement to RNA testing, especially after the second week after symptoms onset [Table 4; (41, 51, 109)]. They have proven to be helpful when nucleic acid tests continue to be negative in suspected patients, possibly because too many days have passed since infection and lower antigen levels mislead results (41, 77). In addition, simultaneous detection of both IgM and IgG can reveal valuable information about the time course of the infection, thus giving useful leads for treatment. In conclusion, combining RNA testing with antibody detection significantly improves diagnosing sensitivity, which is the ultimate goal.

**Table 4:** Comparison of current SARS-CoV-2 current diagnosis approaches reviewed.

**Type of Assay**		**Target type**		**Time needed**	**Site where testing takes place**	**Advantage**		**Limitation**		**Suggested use**	
		**Looks for**	**Targeting at**								
Direct testing	rRT-PCR	Virus replication (active infection)	Virus genome	3–4 h	Laboratory	Highly sensitive and specific		High turnaround condition^*^, requires skilfull personnel and its expensive		COVID-19 diagnosis and screening of contacts among infectious cases	
	Ag-based RDT		Viral antigens	15 min	POC diagnosis	Easy-to-use, low turnaround condition, cheaper		Low sensitivity and specificity. Needs further diagnosis confirmation.		A Large-scale screening diagnostic test	
Indirect testing ELISAs		Host antibody response (active and past infection)	Antibodies^a^	1–3 h	Laboratory	Sample collection supposes a lower exposure risk	Highly sensitive and specific	Time-dependent on the host antibody development	High turnaround condition, requires skilfull personnel and its expensive	Identifying possible human donors for collection of convalescent serum, during vaccine trials and recognizing possible animal hosts for SARS-CoV-2	A complement to RNA testing, especially since the 2nd week after symptoms onset. Immunoglobulins detection reveal information about the time course of the infection.
	CLIAs		Antibodies^b^	1–3 h	Laboratory						
	Ab-based RDT		Antibodies^c^	15 min	POC diagnosis		Easy-to-use, low turnaround condition, cheaper		Low sensitivity and specificity		Screening seroprevalence levels among the population

a *Either IgM or IgG, or both, or IgA, or total antibodies*.

b *Either IgM or IgG, or both, or total antibodies*.

c *Either IgM or IgG, or both*.

* *The turn-around time of rapid tests lasts about an hour*.

## The Importance of the Detection of Both Antibodies

Besides being a complement to RNA testing in the diagnosis of COVID-19, antibody detection using immunoassay tests has many other applications. In the present situation, immunoassays are being used within the context of epidemiological studies to determine which are the seroprevalence levels among the population (41). Furthermore, SARS-CoV-2 based immunoassays are helpful to guide the identification of possible human donors for collecting convalescent serum, which is considered a possible promising treatment (95). Finally, immunoassays may play a crucial role during vaccine trials and in recognizing possible animal hosts for SARS-CoV-2 (41, 88).

The studies conducted so far have revealed a significant correlation between antibody titers and that the clinical severity of the disease remains beyond the second week after illness onset—the higher the antibody titers, the worse the prognosis (41). Moreover, it has been reported that antibody detection rates are lower in younger subjects (5, 74). However, even though the cause is not yet known, and further research is needed, what is clear is that antibody measurement can be a marker of disease severity and may be helpful in treatment decision making.

Moreover, Wu et al. measured SARS-CoV-2 specific neutralizing antibodies (NAbs) among recovered patients and observed that about 30% of subjects developed very low NAbs levels, suggesting the presence of alternatives pathways besides NAbs production against the virus (5).

Despite the recent concern of the WHO in regard to the lack of data demonstrating immunization, and whether immunization protects against SARS-CoV-2 reinfection, a study conducted in China demonstrated that reinfection did not occur in Rhesus macaques after recovering from SARS-CoV-2 infection (110). Finally, due to the recent onset of the pandemic, data concerning how long antibodies last is not yet known (41).

## Discussion

This review was aimed at analyzing the current COVID-19 diagnostic approaches available, with the added difficulties of the recent onset of the pandemic and the huge amount of incoming information. The constant development of new commercial diagnostic tests will subject any conclusion drawn here to obligatorily be revised. In the meantime, even though it is too soon to derive definitive results, the present work intends to be a helpful guide in terms of optimizing diagnosis.

The high degree of homology shared with other human CoVs and the high number of mild COVID-19 cases demonstrate the need for both sensitive and specific diagnostic tests. RT-PCR is currently the most accurate test. Two automatized platforms can currently be used: (1) Integrated platforms which provide a result in 1 h or 1 h and a half, although they cannot process many samples at once, and (2) Integrated platforms which process more than 90 samples at once but the turnaround time is of around 3.5–4 h. Meanwhile, immunoassays, for which time-dependent accuracy is their major inconvenience, are considered as a complement to nucleic acid tests, especially after 14 days since illness onset. Moreover, these tests have many other applications besides diagnosis, as noted along the text. Finally, the primary goal of RDTs is to obtain point-of-care diagnosis, but they lack sensitivity and specificity and need further diagnostic confirmation.

There is a need for sharing findings as well as providing transparent results when testing different diagnostic kits. As mentioned previously, at the time of writing FIND is conducting a generalized evaluation of the commercial kits available, using a standardized independent protocol, in order to provide practical advice based on robust evidence-based results.

## Author Contributions

AM-V, CB-D, and JV contributed to the design of the text and writing. AM-V design the tables. JV design figures. All authors contributed to the article and approved the submitted version.

## Conflict of Interest

The authors declare that the research was conducted in the absence of any commercial or financial relationships that could be construed as a potential conflict of interest.
