# Supplementary material for: Review of the Microbiological Diagnostic Approaches of COVID-19
Source: Front Public Health. 2021 Apr 27;9:592500. doi: 10.3389/fpubh.2021.592500 (PMC8110909; doi:10.3389/fpubh.2021.592500)
Supplement: Supplementary file 1 [file Data_Sheet_1.PDF]

SUPPLEMENTARY DATA

Supplementary Table 1. External studies testing commercially available ELISAs listed on the Foundation for Innovative New Diagnostics (FIND)'s website.

Reference	Test	Company	Test Name	Type of test	Antigen used for detection	Sample type	Gold Standard Method	Number of patients (Confirmed Covid19/ Suspected Covid19/ Healty)	Ab detected	Days from onset	Sensitivity	Specificity	VPP	VPN	Applications	Comments
**(Xiang *et al*. 2020)**	Commercial	**Zhuhai Livzon Diagnostics Inc (China)**	IgG/IgM ELISA kit	Enzyme-linked immunosorbent assay (ELISA)	Recombinant N protein of SARS-CoV-2	Serum	RT-PCR	85/24/60	IgM	Samples picked on different days after onset (more than 13 days and less than 29).	77,3% [a]	100% [a]	100% [a]	80% [a]	Antibody detection can play an important role in diagnosis during the tje middle and later stage of the infection.	In addition, diagnostic value of serological IgM and IgG antibodies was evaluated in 24 suspected COVID-19 pneumonia patients with negative nucleic acid tests.
									IgG		83,3% [a]	95% [a]	94,8% [a]	83,8% [a]		
**(Zhao *et al.* 2020)**	Commercial	**Beijing Wantai Biological Pharmacy Enterprise Co., Ltd (China)**	IgG/IgM ELISA kits	Double-antigens sandwich immunoassay (Ab-ELISA)	receptor binding domain (RBD) of the spike protein of SARS-CoV-2	Plasma	RT-PCR	173/0/0	Total Antibodies (Ab)	Samples collected at different days after symptom onset: 1-7 / 8-14 / 15-39	93,10%	99,1% [b]			Serological test is seen as a complement to RNA detection during the illness course, especially when nucleic acid tests keeps turning negative in suspected patients.	Specificity was determined testing samples collected from healthy indivuals before the SARS-CoV-2's outbreak. Cross-reactivity to other coronaviruses has not been assessed.
				μ-chain capture method (IgM-ELISA)					IgM		82,70%	98,6% [b]				
				Indirect ELISA kit (IgG-ELISA)	Recombinant nucleoprotein				IgG		64,70%	99% [b]				
**(Xiang *et al.* 2020)**	Commercial	**Zhuhai Livzon Diagnostics Inc (China)**	IgG/IgM antibody ELISA kits	IgM capture ELISA	Antigen unspecified	Serum	RT-PCR	63/0/35	IgM	Unspecified	44,44%	100%			Serological test can be used for clinical diagnosis, relieving the huge pressure on diagnosis	Antigen used for detection nor days from onset when samples were collected are not specified. Combined IgM and IgG detection provides a higher sensitivity value. The antibody used in the kit is not only reactive against SARS-CoV-2, but also against SARS-CoV. Therefore, if SARS-IgG is present, a positive result will be obtained.
				IgG indirect ELISA	Recombinant antigen of new coronavirus (unspecified)				IgG		82,50%	100%				
									IgM and/or IgG*		87,30%	100%				
**(Lassaunière *et al.* 2020)**	Commercial	**Beijing Wantai Biological Pharmacy Enterprise Co., Ltd (China)**	Wantai SARS-CoV-2 Ab ELISA	Double-antigen sandwich ELISA	Receptor binding domain (RBD) of the spike protein of SARS-CoV-2	Serum or plasma	RT-PCR	30/0/82	Total Antibodies (Ab)	Samples collected at different days after sympton onset: 7 to 13 days, 14 to 20 days, >21 days	93,00%	100%	100%	98%	Identifying individuals with an adaptative immune response to SARS-CoV-2, indicating recent or prior infection. Besides diagnosis, it can be used in sero-epidemiological and vaccine development studies.	
	Commercial	**Euroimmun Medizinische Labordiagnostika (Germany)**	Anti-SARS-CoV-2 IgG ELISAs	Two separate semi-quantiative ELISAs	SARS-CoV-2 spike protein subunit 1 (S1)				IgG		67,00%	96%	87%	89%		Show cross-reactivity to serum containing antibodies to human coronavirus HKU1 and to two serum samples containing adenovirus antibodies
			Anti-SARS-CoV-2 IgA ELISAs						IgA		93,00%	93%	82%	97%		Show cross-reactivity to serum containing antibodies to more than one respiratory virus, to adenovirus antibodies and dengue virus antibodies.

Reference	Test	Company	Test Name	Type of test	Antigen used for detection	Sample type	Gold Standard Method	Number of patients (Confirmed Covid19/ Suspected Covid19/ Healty)	Ab detected	Days from onset	Sensitivity	Specificity	VPP	VPN	Applications	Comments
(Liu *et al.* *2020*)	Commercial	**Zuhai Lizhu Reagent Co., Ltd. (China)**	2019-nCoV IgG/IgM Antibody detection kit	Enzyme-linked immunosorbent assay (ELISA)	Recombinant nucleocapsid (rN) protein	Serum	RT-PCR	214/0/100	IgM	Median number of days post onset is 15 days (range from 0 to 55 days)	68,20%	100%			The author recommends rN- and rS-based ELISAs as an screening method for COVID-19 cases. It can be used as a large-scale confirmation or exclusion of subjects.	Comparison of two different antigen-based ELISAs revealed that rS-based ELISA happen to be more sensitive than rN-based when detecting IgM antibodies.
									IgG		70,10%	100%				
									IgM and/or IgG*		80,40%	100%				
	Commercial	**Beijing Hotgen Biotech Co., Ltd.**	2019-nCoV IgG/IgM Antibody detection kit	Enzyme-linked immunosorbent assay (ELISA)	Receptor Binding Domain (RBD) of the recombinant S polypetide (rS)				IgM		77,10%	100%				
									IgG		74,30%	100%				
									IgM and/or IgG*		82,20%	100%				
(Lin *et al.* *2020*)	Commercial	**Guangzhou Darui Biotechnology Co., Ltd. (China)**	SARS-CoV-2 IgM Antibody Detection Kit (ELISA)	Enzyme-linked immunosorbent assay (ELISA)	SARS-CoV-2 specific Nucleocapsid Protein	Serum	RT-PCR	65/0/64	IgM	Samples collected at different days after onset	46,10%	82%				In this study they compared the commercially available Darui Biotechnology ELISA to an in-house developed CLIA. This second one proved to have significantly higher sensitivity. The sensitivity difference may be attributed to intrinsec method characteristics or to differences in serum titers during the incubation step.
			SARS-CoV-2 IgG Antibody Detection Kit (ELISA)						IgG		23%	100%				
(Adams *et al.* *2020*)	Commercial (In development)	**Mologic Ltd. (UK)**	COVID-19 IgG-ELISA	Enzyme-linked immunosorbent assay (ELISA)	Nucleocapsid Protein and Spike Protein Subunit 2 (S2)	Serum	RT-PCR	124/90/564	IgG	Samples collected at different days after onset, ranging from day 0 to day 42	88% [a]	97% [b]			Mologic products are thought to be used in COVID-19 diagnosis, contact tracing and epidemiological studies.	ELISA negative samples were on average collected sooner (3 days) than ELISA positive samples
(Lou *et al.* *2020*)	Commercial	**Beijing Wantai Biological Pharmacy Enterprise Co., Ltd., (China)**	ELISA-Ab	Double-antigens sandwich immunoassay (Ab-ELISA)	Receptor binding domain (RBD) of the SARS-CoV-2 Spike protein	Plasma	RT-PCR	80/0/300	Total Antibodies (Ab)	Samples collected at different days after onset: 0 to 7 days, 8 to 14 days, 15 to 29 days	97,5%	100%			Immunoassays are seen as a complement to RNA testing, being helpful to evaluate the time course of the infection, as well as the immunity status.	
			ELISA-IgM	µ-chain capture method immunossay (IgM-ELISA)					IgM		92,5%	100%				
			ELISA-IgG	Indirect ELISA (IgG-ELISA)	Recombinant nucleoprotein of SARS-CoV-2				IgG		88,8%	100%				

Supplementary Table 2. External studies testing commercially available LFIAs listed on the Foundation for Innovative New Diagnostics (FIND)'s webpsite.

Reference	Test	Company	Test Name	Type of test	Antigen used for detection	Sample type	Gold Standard Method	Number of patients (Confirmed Covid19/ Suspected Covid19/ Healty)	Ab detected	Days from onset	Sensitivity	Specificity	VPP	VPN	Applications	Comments
(Imai *et al*. 2020)	Commercial	**Artron Laboratories Inc. (Canada)**	One Step Novel Coronavirus (COVID-19) IgM/IgG Antibody Test	Lateral Flow Immunoassays (LFIA) based on immunochromatography	Unspecified	Serum	RT-PCR	112/0/48	IgM	Samples collected at different days after onset: within 1 week, 1-2 weeks, and >2 weeks	43,20%	98%			The author recommends that IC assay should not be used alone for initial diagnostic testing due to its low sensibility. However it could be used for epidemiological studies of the seroprevalence of antibodies against SARS-CoV-2	1 specimen from non-COVID-19 serum specimen collected before SARS-CoV-2 infection showed a false-positive result for IgM antibody. The patient happen to be diagnosticated with Sjogren's syndrome and rheumatoid arthritis.
									IgG		14,40%	100%				
(Paradiso *et al.* 2020)	Commercial	**Jiangsu Medomics Medical Technologies (China)**	SARS-CoV-2 rapid IgG-IgM Viva-DiagTM Test combined antibody test kit	Lateral Flow Immunoassays (LFIA)	Receptor-Binding Domain of the COVID-19 spike protein	Whole blood, serum and plasma	RT-PCR	191/0/0	IgM-IgG	Samples collected at different days from the onset of symptoms: 0-5, 6-8, 9-10, 11-15, >15	30%	89%			The author recommends that the test should not be used for the diagnosis. However it can be used for epidemiological studies and detection of previous exposure to the virus in currently healthy people.	13 patients (7%) had positive serological tests and instead negative RT-PCR results. These were not considered false negatives but instead recovered patients. At the present time there is a trial going on, registered in ClinicalTrial.gov (NCT04316728), investigating seroconversion of COVID-19 IgG/IgM in healthy subjects using VivaDiag device.
(Li *et al. 2020*)	Commercial	**Jiangsu Medomics Medical Technologies (China)**	SARS-CoV-2 rapid IgG-IgM combined antibody test kit	Lateral Flow Immunoassays (LFIA)	Receptor-Binding Domain of the COVID-19 spike protein	Whole blood, serum and plasma	RT-PCR	397/0/128	IgM-IgG	Unspecified	88,66%	90,63%			Because the test detecs both IgM and IgG, it can be used for monitoring the infection course	Underlines that detection sensibility is higher when performed IgG-IgM combined antibody test than individual IgG or IgM antibody test
(Xiang *et al. 2020*)	Commercial	**Zhuhai Livzon Diagnostics Inc (China)**	novel coronavirus IgG/IgM antibody GICA kits	Lateral Flow Immunoassays (LFIA)	Unspecified	Whole blood, serum and plasma	RT-PCR	91/0/35	IgM	Unspecified	57,10%	100%			Serological test can be used for clinical diagnosis, relieving the huge pressure on diagnosis	Antigen used for detection nor days from onset when samples were collected are not specified. Combined IgM and IgG detection provides a higher sensitivity value. The antibody used in the kit is not only reactive against SARS-CoV-2, but also against SARS-CoV. Therefore, if SARS-IgG is present, a positive result will be obtained.
									IgG		81,30%	100%				
									IgM-IgG		82,40%	100%				

Reference	Test	Company	Test Name	Type of test	Antigen used for detection	Sample type	Gold Standard Method	Number of patients (Confirmed Covid19/ Suspected Covid19/ Healty)	Ab detected	Days from onset	Sensitivity	Specificity	VPP	VPN	Applications	Comments
(Lassaunière *et al.* 2020)	Commercial	**Dynamiker Biotechnology (China)**	2019-nCOV IgG/IgM Rapid Test	Lateral Flow Immunoassays (LFIA)	Unspecified	Whole blood, serum and plasma	RT-PCR	30/0/82	IgM-IgG	Samples collected at different days after symptom onset: 7 to 13 days, 14 to 20 days, >21 days	90%	100%	100%	89%	Identifying individuals with an adaptative immune response to SARS-CoV-2, indicating recent or prior infection. Besides diagnosis, it can be used in sero-epidemiological and vaccine development studies.	
	Commercial	**CTK Biotech (USA)**	One site™ COVID-19 IgG/IgM Rapid Test	Lateral Flow Immunoassays (LFIA)	Unspecified				IgM-IgG		90%	100%	100%	89%		
	Commercial	**AutoBio Diagnostics (China)**	Anti-SARS-CoV-2 Rapid Test	Lateral Flow Immunoassays (LFIA)	Unspecified				IgM-IgG		93%	100%	100%	91%		
	Commercial	**Artron Laboratories Inc. (Canada)**	Coronavirus Diseases 2019 (COVID-19) IgM/IgG Antibody Test	Lateral Flow Immunoassays (LFIA)	Unspecified				IgM-IgG		83%	100%	100%	74%		
(Cassaniti *et al.* 2020)	Commercial	**Vivacheck Biotech (China)**	VivaDiag COVID-19 IgM/IgG Rapid Test	Lateral Flow Immunoassays (LFIA)	Unspecified	Serum or whole blode sample	RT-PCR	30/50/30	IgM-IgG	Unspecified	18,4% [c]	91,7% [c]	87,5% [c]	26,2% [c]	Author does not recommend using it with patients at their first access at emergency room department.	Results disagree with those reported by Li Z et *al.* Previous study, in which VivaDiag Rapid Test was also assessed
(Pérez-García *et al.* 2020)	Commercial	**Hangzhou Alltest Biotech Co., Ltd (China)**	AllTest COVID-19 IgG/IgM	Lateral Flow Immunoassays (LFIA)	Unspecified	Serum or plasma samples	Pneumonia of unknown etiology and negative PCR	55/63/55	IgM and/or IgG *	Median days after symptom onset was 17 days (13-22)	88,9% [d]	100% [d]			Reliable tool to diagnose from 14 days of onset of symptoms. Also in subjects whose PCR results has turn out to be negative	Specificity and Sensitivity values are calculated based on test performance on the Group of subjects with pneumonia of unknown etiology and negative PCR. In addition, specificity was calculated based on 55 healthy subjects whose serum had been collected previous to SARS-CoV-2 outbreak, obtaining 100% Specificity. In addition, sensibility was calculated based on 55 RT-PCR confirmed COVID-19 cases, obtaining a lower specificity value: 47% (median days after symtpom onset was 11 days).

Reference	Test	Company	Test Name	Type of test	Antigen used for detection	Sample type	Gold Standard Method	Number of patients (Confirmed Covid19/ Suspected Covid19/ Healty)	Ab detected	Days from onset	Sensitivity	Specificity	VPP	VPN	Applications	Comments
(Pan *et al.* *2020*)	Commercial	**Zuhai Livzon Diagnostics Inc (China)**	Colloidal Gold-based immunochrom-atographic (ICG) strip	Lateral Flow Immunoassays (LFIA)	Unspecified	Whole blood, serum and plasma	RT-PCR	86/37/22	IgM	Samples collected at different days from the onset of symptoms: 1-7, 8-14, >15	55,8% [a]				Used as a supplementary diagnose tool, especially since the second week after onset of symptoms	The study says that no specificity analysis was performed as the study was conducted in Wuhan and there was no certainty to select uninfected cases for the negative control.
									IgG		54,7% [a]					
									IgM and/or IgG *		68,6% [a]					Combining IgM and IgG detection results maximizes testing efficacy
(Lou *et al.* *2020*)	Commercial	**Beijing Wantai Biological Pharmacy Enterprise Co., Ltd., (China)**	LFIA-Ab	Double-antigens sandwich lateral flow immunoassay	Receptor binding domain (RBD) of the SARS-CoV-2 Spike protein	Plasma	RT-PCR	80/0/300	Total Antibodies (Ab)	Samples collected at different days after onset: 0 to 7 days, 8 to 14 days, 15 to 29 days	97,5%	95,2%			Immunoassays are seen as a complement to RNA testing, being helpful to evaluate the time course of the infection, as well as the immunity status.	
			LFIA-IgM	μ-chain capture method lateral flow immunossay					IgM		88,8%	98,1%				
			LFIA-IgG	Indirect lateral flow immunoassay	Recombinant nucleoprotein of SARS-CoV-2				IgG		86,3%	99,5%				

Supplementary Table 3. External studies testing commercially available CLIAs listed on the Foundation for Innovative New Diagnostics (FIND)'s website.

Reference	Test	Company	Test Name	Type of test	Antigen used for detection	Sample type	Gold Standard Method	Number of patients (Confirmed Covid19/ Suspected Covid19/ Healty)	Ab detected	Days from onset	Sensitivity	Specificity	VPP	VPN	Applications	Comments
(Jin *et al.* 2020)	Commercial	**Shenzhen YHLO Biotech Co., Ltd (China)**	iFlash-SARS-CoV-2 IgM	Chemiluminesc-ence Enzyme Immunoassays (CLIA)	N protein and S protein	Serum	RT-PCR	43/0/33	IgM	The median duration from symptom onset to serological testing was 16 days (9-20 days).	48,10%	100%	100%	70,20%	Effective immunoassay for diagnosis needs.	Time to nucleic acid testing and serological testing is different, being 3 and 18 days respectively
			iFlash-SARS-CoV-2 IgG						IgG		88,90%	90,90%	88,90%	90,90%		
(Zhang *et al. 2020*)	Commercial	**Shenzhen Yahuilong Biotechnology Co Ltd (China)**	Chemiluminescent detection kit	Chemiluminesc-ence Enzyme Immunoassays (CLIA)	2019-nCoV Spike protein (S) and Nucleocapsid protein antigen (N)	Serum	RT-PCR	3/0/733	IgM	days after symptom onset: 10/7/12 days	100%	97,33%	33,33%	100%	It is seen as a useful compelement to nucleic acid testing.	Only 3 confirmed COVID-19 cases were included in the study. A large cohort is need to verify sensibility and specificity values. Shows cross-reactivity to a small sample of influenza A and B, Adenovirus and mycoplasma pneumonia cases.
									IgG		100%	99,56%	75%	100%		
(Lou *et al.* 2020)	Commercial	**Xiamen InnoDx Biotech Co., Ltd., (China)**	CMIA-Ab	Double-antigens sandwich Chemiluminesn-ce microparticle immunoassays (CMIA)	Receptor binding domain (RBD) of the SARS-CoV-2 Spike protein	Plasma	RT-PCR	80/0/300	Total Antibodies (Ab)	Samples collected at different days after onset: 0 to 7 days, 8 to 14 days, 15 to 29 days	96,30%	99,30%			Immunoassays are seen as a complement to RNA testing, being helpful to evaluate the time course of the infection, as well as the immunity status.	
			CMIA-IgM	μ-chain capture method Chemiluminesn-ce microparticle immunoassays (CMIA)					IgM		86,30%	99,30%				

Supplementary Table 4. External studies testing commercially available antigen-based rapid diagnostic tests (Ag-based RDTs) listed on the Foundation for Innovative New Diagnostics (FIND)'s website**.**

Reference	Test	Company	Test Name	Type of test	Antigen detected	Sample type	Gold Standard Method	Number of patients (Confirmed Covid19/ Suspected Covid19/ Healty)	Days from onset	Sensitivity	Specificity	VPP	VPN	Applications	Comments
(Porte *et al*. 2020)	Commercial	**Bioeasy Biotechnology Co., Shenzen (China)**	Diagnostic kit for 2019-Novel Coronavirus Ag Test	Fluorescence Immunochromatographic Assay	SARS-Cov-2 Nucleocapsid protein	Nasopharyngeal and Oropharyngeal swabs	RT-PCR	82/0/45	Samples collected during the first week of the disease	93,90%	100%	100%	99,40%	Antigen tests can be more useful in the first phase of the infection course.	A biosafety cabinet is required for handling the samples. Positive and negative predictive values are extrapolated.
(Diao *et al.* 2020)	Commercial	**Bioeasy Biotechnology Co., Shenzen (China)**	Bioeasy 2019-nCoV Ag Fluorescence Rapid Test Kit	Fluorescence Immunochromatographic Assay	SARS-Cov-2 Nucleocapsid protein	Nasopharyngeal swab	RT-PCR	208/31	Unspecified	68%	100%	100%	32%	Used for early-diagnosis, as well as for large-scale screening	N antigen detection of SARS-CoV-2 was perfomed in urine samples in paralel with nucleic acid test of nasopharyngeal swabs, obtaing a sensitivity value of 73,6%
										98%	100%	100%	97%		

[a] Sensitivity, specificity, VPP and VPN values were calculated in the SARS-CoV-2 RT-PCR confirmed group of subjects.

[b] Specificity is assessed testing samples collected from healthy individuals prior to the SARS-CoV-2 outbreak.

[C] Sensitivity, specificity, VPP and VPN values were calculated in the SARS-CoV-2 RT-PCR confirmed group of suspected subjects.

[d] Sensitivity and specificity values assessed in the group of suspected Covid-19 subjects (with pneumonia of unknown etiology and negative PCR).

*Positive if either of two markers was positive.

If samples were collected at different times of onset, the sensitivity value provided is the total sensitivity.
